# mRNA profiling of a well-differentiated G1 pancreatic NET correlates with immunohistochemistry profile: a case report

**DOI:** 10.1186/s12876-021-01705-9

**Published:** 2021-04-27

**Authors:** Abhirami Venugopal, Jessie Gillick-Walker, Agnes Michalczyk, Mustafa Khasraw, M. Leigh Ackland

**Affiliations:** 1 Centre for Cellular and Molecular Biology, School of Life and Environmental Sciences, Deakin University, Burwood, VIC 3125 Australia; 2 Duke University Medical Center, Durham, NC 27710 USA

**Keywords:** Neuroendocrine tumours (NET), Biomarkers, MRNA, QRT-PCR, Immunohistochemistry

## Abstract

**Background:**

Neuroendocrine neoplasms (NENs) are a complex group of tumours that occur in many organs. Routinely used IHC markers for NEN diagnosis include CgA, synaptophysin, Ki67 and CD56. These have limitations including lack of correlation to clinical outcomes and their presence in non-tumour tissue. Identification of additional markers and more quantitative analyses of tumour tissue has the potential to contribute to improved clinical outcomes. We used qRT-PCR to profile the expression levels of a panel of markers in tumour and matched non-tumour tissue from a patient with a G1 pancreatic neuroendocrine tumour. Differences in mRNA levels between tumour and non-tumour tissue were compared with IHC analyses of the same sample.

**Case presentation:**

An elderly man presented with lower abdominal pain for 6 months. Histological analysis identified a low grade, well differentiated pancreatic endocrine neoplasm. Twenty-seven tumour markers for neuroendocrine status, proliferation, stem cell phenotype, angiogenesis, epithelial to mesenchymal transition, cell adhesion, differentiation and tumour suppression were selected from previous studies and mRNA levels of these markers were measured in tumour and adjacent non-tumour tissue sample using qRT-PCR. IHC was carried out on the same tissue to detect the corresponding marker proteins. Of the markers analysed, seven showed higher mRNA levels in tumour relative to non-tumour tissue while thirteen had lower expression in tumour relative to non-tumour tissue. Substantial differences in mRNA levels were a gain of CgA, CD56, β-catenin, CK20, PDX1 and p53 and loss of Ki67, PCAD, CK7, CD31, MENA, ECAD, EPCAM, CDX2 and CK6. Comparison of qRT-PCR data with IHC showed correlation between fifteen markers.

**Conclusion:**

Our study is unique as it included matched controls that provided a comparative assessment for tumour tissue analysis, whereas many previous studies report tumour data only. Additionally, we utilised qRT-PCR, a relatively quantitative diagnostic tool for differential marker profiling, having the advantage of being reproducible, fast, cheap and accurate. qRT-PCR has the potential to improve the defining of tumour phenotypes and, in combination with IHC may have clinical utility towards improving tumour stratification or distinguishing tumour grades. The results need to be validated with different grades of NENs and related to clinical outcomes.

**Supplementary Information:**

The online version contains supplementary material available at 10.1186/s12876-021-01705-9.

## Background

Neuroendocrine neoplasms (NENs) are a heterogeneous group of malignancies that can occur in all organs of the body, with the gastrointestinal system being a common site of localisation. Within the GI tract, the most common sites are the small intestine, rectum, colon, stomach, pancreas and appendix [1]. NENs express markers of neuroendocrinology differentiation and may secrete a range of peptides that can cause hormonal symptoms [2] Some features of neuroendocrine differentiation are common to all NENs while others may be site-specific [3]. This has caused confusion in the classification of NENs because morphologically similar tumours can be found in different organs.

Gastrointestinal NENs are clinically challenging in their management. Some patients present with circulating levels of biologically active peptides including circulating chromogranin A and serotonin while others, termed non-functional NENs may have no specific clinical indicators but present with local symptoms including obstruction and bleeding [4]. Non-functional pancreatic tumours account for 85% of all pancreatic tumours and have a significantly worse outcome than functional tumours and they may only present after metastasis [5].

Histological morphology and immunohistochemical criteria are routinely used to diagnose NENs. Established diagnostic immunohistochemical markers include Ki-67 a marker of proliferation, chromogranin A (CgA), synaptophysin, and CD56 [6, 7]. Additional markers used including CDX2, a transcription factor required for intestinal differentiation [8, 9], cytokerations, [10] and the transcription factor PDX-1 that is required for pancreatic ductal and islet cell development [11]. It is clear that the complexity of a tumour cannot be defined by a small set of markers but on the other hand there is a limit to the number of tests that can be carried out for routine diagnostic purposes.

Based on the 2019 WHO classification [12], NENs are classified into three grades: Grade 1 (low grade) with a mitotic rate < 2 and Ki‐67 < 3% (NET, G1), Grade 2 (intermediate) with a mitotic rate < 2 and Ki‐67 3‐20%, (NET, G2) and Grade 3 (high) with both mitotic rates and Ki‐67 > 20% (NET, G3). NECs are small cell type or large cell type, both poorly differentiated and with both mitotic rate and Ki‐67 > 20%.

In a study of 200 patients with pancreatic endocrine carcinoma, the Ki-67 index was the major risk factor in relation to disease progression [13]. While the Ki67 index is a WHO standard indicator of tumour proliferation and predictor of disease outcome, significant inter and intra-lab variabilities in Ki-67 grading due to technical variations and observer differences have been demonstrated [14]. The 2010 International Ki67 in Breast Cancer Working Group acknowledged the enormous variation in analytical practice that limits the value of Ki67 [15].

A lack of reliable predictive and prognostic markers in neuroendocrine neoplasms on which to base therapeutic choices was identified by the ENETs [16, 17]. Single markers cannot define the numerous cellular changes associated with cancer. Analyses of additional molecules whose functions are known to be altered in cancer including markers of proliferation, metabolic activity, invasive potential, metastatic propensity would provide possibilities for stratification of tumours that may inform treatment.

The aims of this study were to use RT-qPCR to measure mRNA levels of twenty-seven markers in a pancreatic G1 NET tumour relative to matched non-tumour tissue, to obtain a marker profile that could have clinical utility towards improving stratification of G1 pancreatic tumours or distinguishing tumour grades.

### Case presentation

A 66 year-old gentleman presented to his chiropractor with lower back pain having been asymptomatic for the previous 6 months. An ultrasound scan of his kidneys and a subsequent CT KUB + Abdo/Pelvis showed two solid lesions at the mid pole and lower pole of the right kidney and one heterogeneous solid lesion at the lower pole of the left kidney. It also showed a lesion in the pancreatic head measuring 3 cm with a dilated pancreatic duct and two small lymph nodes in the periportal region.

A CT scan of the chest was done to complete staging and this showed a 3 mm nodule in the inferior segment of the lingula. This was indeterminate and too small to be characterized. Apart from that, his CT of the abdomen and pelvis was reviewed and this confirmed two solid lesions in the right kidney and also one in the left kidney. The pancreatic head lesion was also identified with the two small lymph nodes in the peri-portal region. In terms of blood tests, his tumour markers were all within the normal limits. This included a CA19.9 of 32, CEA 1.8, PSA < 0.1 and alpha feta protein of 4.

An open distal pancreatectomy was carried out. After two years there was no clinical, radiological or biochemical evidence of recurrence of the neuroendocrine tumour.

### Histopathological findings

Histopathological analysis identified a well differentiated pancreatic endocrine neoplasm, low grade which was encapsulated on the external surfaces. However, elsewhere within the pancreas, tumour had infiltrated into the adjacent exocrine gland. The tumour had an organoid growth pattern with tubular, alveolar and trabecular groupings separated by a fibrous stroma. Cells showed mild nuclear pleomorphism with small nucleoli and gritty chromatin with abundant granular cytoplasm. Mitoses were not seen in 50 HPF and no necrosis was present. Ki-67 staining was variable with generally less than 2% staining positive. No perineural or lymphovascular space invasion was identified. The resections margins were free of tumour with 15 mm of clearance at the proximal pancreatic margin and not present on the inked external surface which was separated from the tumour by 1 mm thick fibrous capsule. A diagnosis of well differentiated pancreatic endocrine neoplasm, low grade pT2NX, was made.

Regions of patient tissue showing normal pancreatic morphology were classed as non-tumour and regions where tissue was disorganised, containing cells with irregular nuclei and heterogeneity of size were classified as tumour tissue [3].

### mRNA marker analysis

Twenty-seven markers previously associated with a range of carcinoma characteristics with purported preventive or prognostic value were selected for the current study. Table 1 lists these markers and their characteristics including neuroendocrine-specific markers and those that are associated with cancer phenotypes including markers for proliferation, stem cell phenotype, angiogenesis, EMT, cell adhesion, differentiation and tumour suppression.

**Table 1:** List of markers and associated characteristics

Categories	Markers
Neuroendocrine	Chromogranin A (CgA), synaptophysin, neuron specific enolase (NSE), CD56
Proliferation	Ki-67, PCNA, DAXX
Stem Cell	CD24, CD44, CD49 (Integrin alpha-6)
Angiogenesis	CD31, CD45
Epithelial to Mesenchymal Transition (EMT)	Vimentin, β-catenin, MENA
Cell Adhesion	Laminin, E-cadherin (ECAD), P-cadherin (PCAD), EpCAM, β-β-catenin, CD56, MENA, CD49
Differentiation	CDX2, PDX1, CD14, CK6, CK7, CK13, CK20
Tumour suppression	P53

**Fig. 1:**
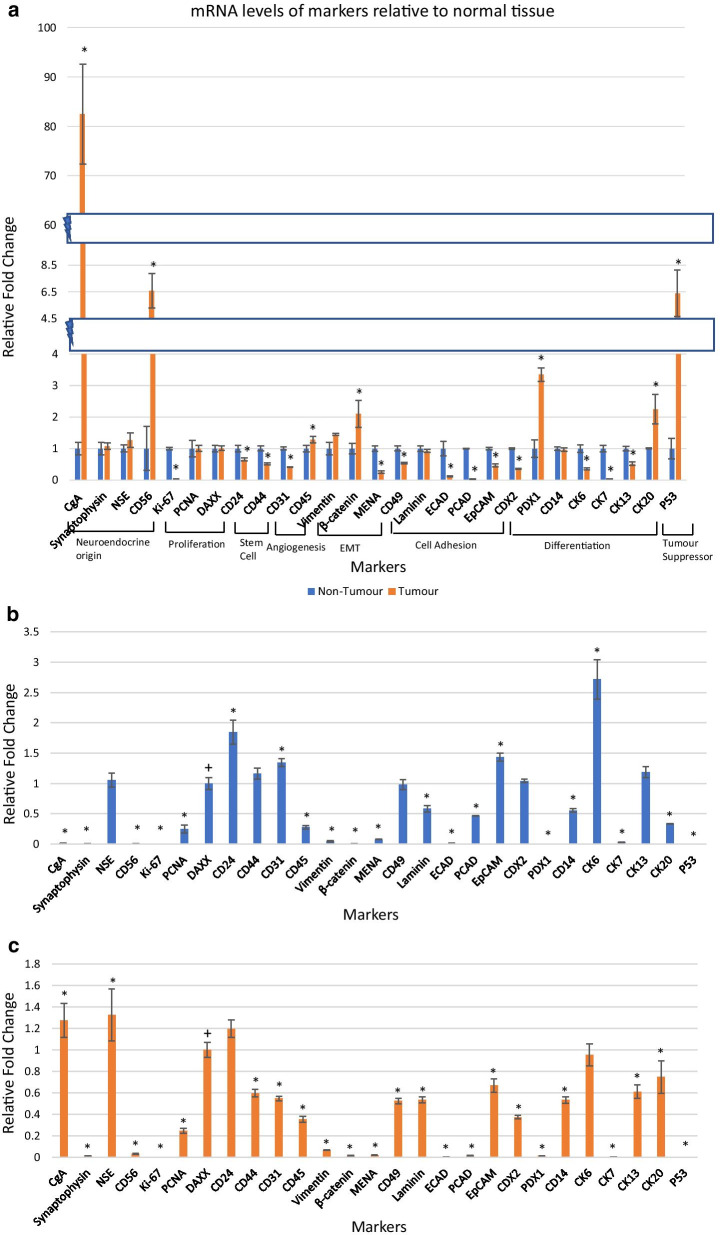
Relative fold change in marker mRNA expression levels using qRT-PCR. * represents the markers that were significantly different in tumour relative to normal. **a** mRNA levels of markers in tumour tissue relative to non-tumour tissue; **b** mRNA levels of markers in non-tumour tissue relative to DAXX; **c** mRNA levels of markers in tumour tissue relative to DAXX. *Represents the markers that were significantly different relative to DAXX expression. +Represents DAXX, used as a control

mRNA transcript levels of each marker were measured in tumour relative to matched non-tumour tissue (Additional file 1). The relative expression of markers was first expressed as fold change calculated using β-actin as the endogenous control. Tumour marker mRNA levels were then expressed relative to the control non-tumour tissue which was given a mRNA expression value of one. Of the twenty-seven markers, mRNA levels of twenty were significantly different in tumour compared to the non-tumour tissue (Fig. 1a). Higher expression levels in tumour relative to non-tumour tissue of > tenfold were found for CgA, 2—tenfold higher mRNA levels were found for CD56, β-catenin, PDX1, CK20, and P53 and 1—twofold higher mRNA levels were found in CD45 tumour tissue compared with the non-tumour tissue. mRNA levels of Ki-67, CD24, CD44, CD31, MENA, CD49, ECAD, PCAD, EpCAM, CDX2, CK6, CK7, CK13, were significantly decreased in tumour tissue relative to non-tumour tissue. NSE and vimentin were higher in tumour relative to non-tumour tissue, but not significantly. The markers synaptophysin, PCNA, DAXX, laminin and CD14 had similar expression levels in tumour and non-tumour tissue. Table 2 summarises the mRNA transcript levels of each marker in tumour tissue relative to non-tumour tissue.

**Table 2:** qPCR analysis of the expression levels of 27 markers in tumour tissue compared to non-tumour, using β-actin as endogenous control

Marker	Expression level
CgA	+ + +*
synaptophysin	=
NSE	+
CD56	+ +*
Ki-67	− − −*
PCNA	=
DAXX	=
CD24	−*
CD44	−*
CD31	− −*
CD45	+*
Vimentin	+
β-catenin	+ +*
MENA	− −*
CD49	−*
Laminin	=
ECAD	− −*
PCAD	− − −*
EpCAM	− −*
CDX2	− −*
PDX1	+ +*
CD14	=
CK6	− −*
CK7	− − −*
CK13	−*
CK20	+ +*
P53	+ +*

+ and − indicates whether marker mRNA transcript levels are higher or lower in the tumour tissue compared to the matched no-tumour tissue. *Represents significant changes (p < 0.05)

−: 1–twofold change lower compared to normal; − −: 2–tenfold change lower compared to normal; − − −: > tenfold change lower compared to normal; +: 1–twofold change higher relative to normal; + +: 2–tenfold change higher relative to normal; + + +: > tenfold change relative to normal; =: similar in tumour and non-tumour

The mRNA levels of markers were compared relative to each other for both tumour and non-tumour tissue. This provided a tissue-specific marker profile for tumour and non-tumour tissue. DAXX was selected for standardisation of mRNA levels as its expression was similar in tumour and non-tumour tissue. DAXX was assigned a value of 1. The relative expression level of each marker in non-tumour tissue relative to DAXX is shown in Fig. 1b. CD24, CD44, CD31, EpCAM and CK6 were expressed significantly higher than DAXX in non-tumour tissue while CgA, synaptophysin, CD56, Ki-67, PCNA, CD45, vimentin, β-catenin, MENA, laminin, ECAD, PCAD, PDX1, CD14, CK7, CK20 and P53 had significantly lower expression levels relative to DAXX in non-tumour tissue. In tumour tissue, CgA and NSE mRNA levels were significantly greater than DAXX while synaptophysin, CD56, Ki67, PCNA, CD44, CD31, CD45, vimentin, β-catenin, MENA, CD49, laminin, ECAD, PCAD, EPCAM, CDX2, PDX1, CD14, CK7, CK13, CK20 and p53 had mRNA levels that were significantly lower than DAXX (Fig. 1c).

### Immunohistochemical analysis

**Fig. 2:**
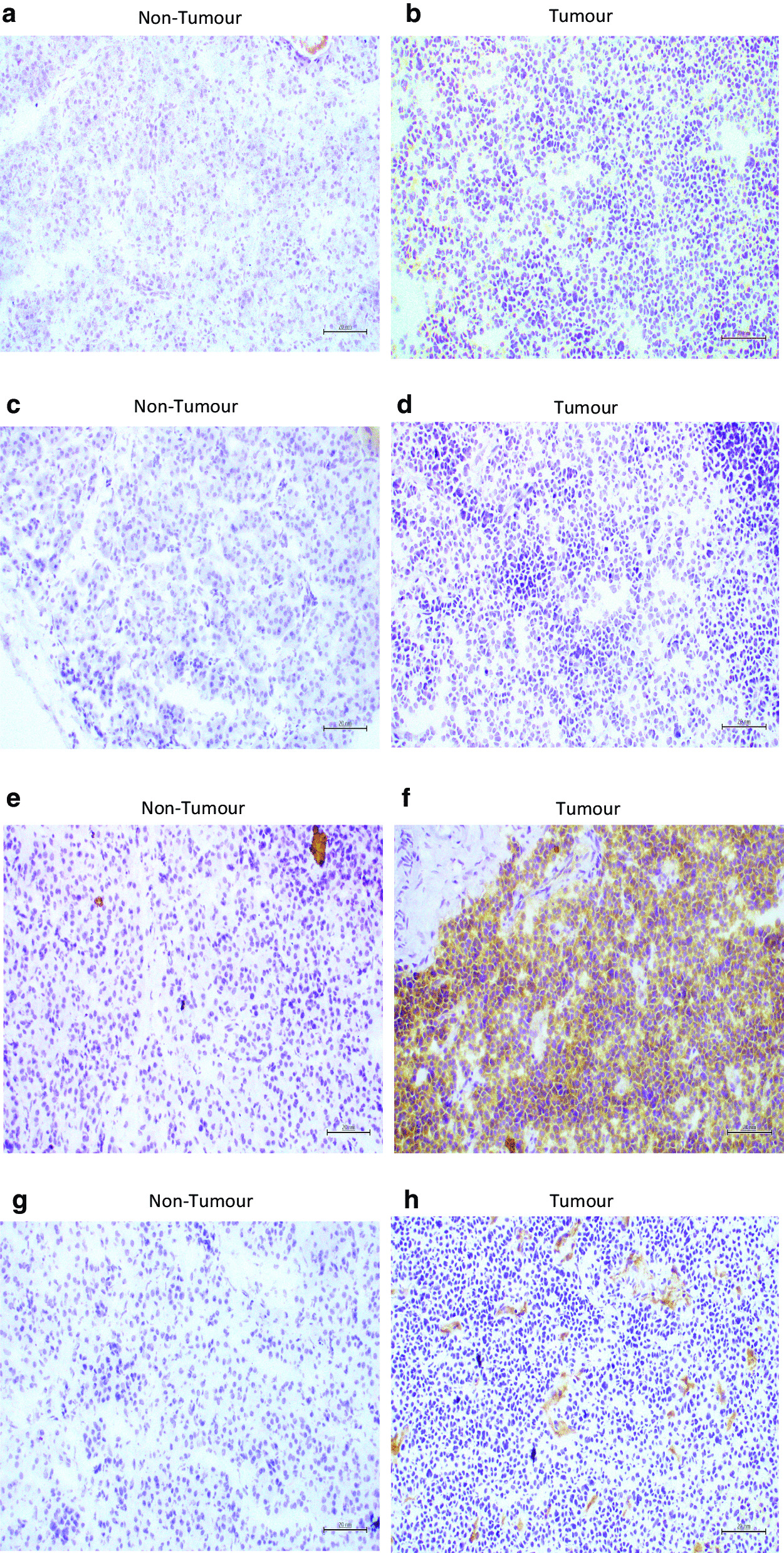
Immunohistochemistry images showing expression levels of CgA (**a**, **b**), PCNA (**c**, **d**), CD24 (**e**, **f**) and CD31 (**g**, **h**) in pancreatic non-tumour and tumour tissue sections. (Scale bars = 20 µM)

Immunohistochemical analysis using an Olympus BX43 light microscope fitted with a 20X/0.40Ph1 lens (PanCN lens) and Olympus DP software was carried out for each of the twenty-seven markers in tumour and non-tumour tissue (Additional file 1). Images were acquired at a resolution of 1920 X 1080. The IHC data is summarised in Table 3. CgA staining was absent in non-tumour tissue but many cells in the tumour tissue were stained (Fig. 2a, b). Synaptophysin staining was absent in both non-tumour and tumour tissue. NSE showed no staining in non-tumour tissue and strong staining in most cells in tumour tissue. CD56 showed < 10% stained cells in non-tumour tissue and staining of 50% of tumour tissue cells. Markers of proliferation Ki-67 and PCNA (Fig. 2c,d) were absent in non-tumour cells and strong nuclear staining was observed in few cells of the tumour tissue. DAXX staining was absent in non-tumour tissue and present in most cells of the tumour tissue.

**Table 3:** Immunohistochemistry analysis of the expression levels of 27 markers in tumour tissue compared to non-tumor

Marker	Expression in Non-Tumour Tissue	Expression in Tumour Tissue
CgA	−	+ + +
synaptophysin	−	−
NSE	−	+ + +
CD56	+	+ +
Ki-67	−	+
PCNA	−	+
DAXX	−	+ + +
CD24	−	+ + +
CD44	+	+ + +
CD31	−	+
CD45	−	+ + +
Vimentin	−	+ +
β-catenin	−	+ + +
MENA	+	+
CD49	+	+
Laminin	+ + +	+
ECAD	+	+ +
PCAD	−	−
EpCAM	+	+
CDX2	−	−
PDX1	+ +	+ + +
CD14	+ +	+
CK6	+ +	+
CK7	+ +	−
CK13	+ +	−
CK20	−	+ +
P53	−	+ +

^*^represents a similar expression pattern as obtained with mRNA analysis. + and − represent the increase and decrease in expression of marker in tumour tissue in comparison to the normal sample

−: Absence of marker expression; + :1- 25% field positive for marker expression; + + : 25–50% field positive for marker expression; + + + : > 50% field positive for marker expression

**Fig. 3:**
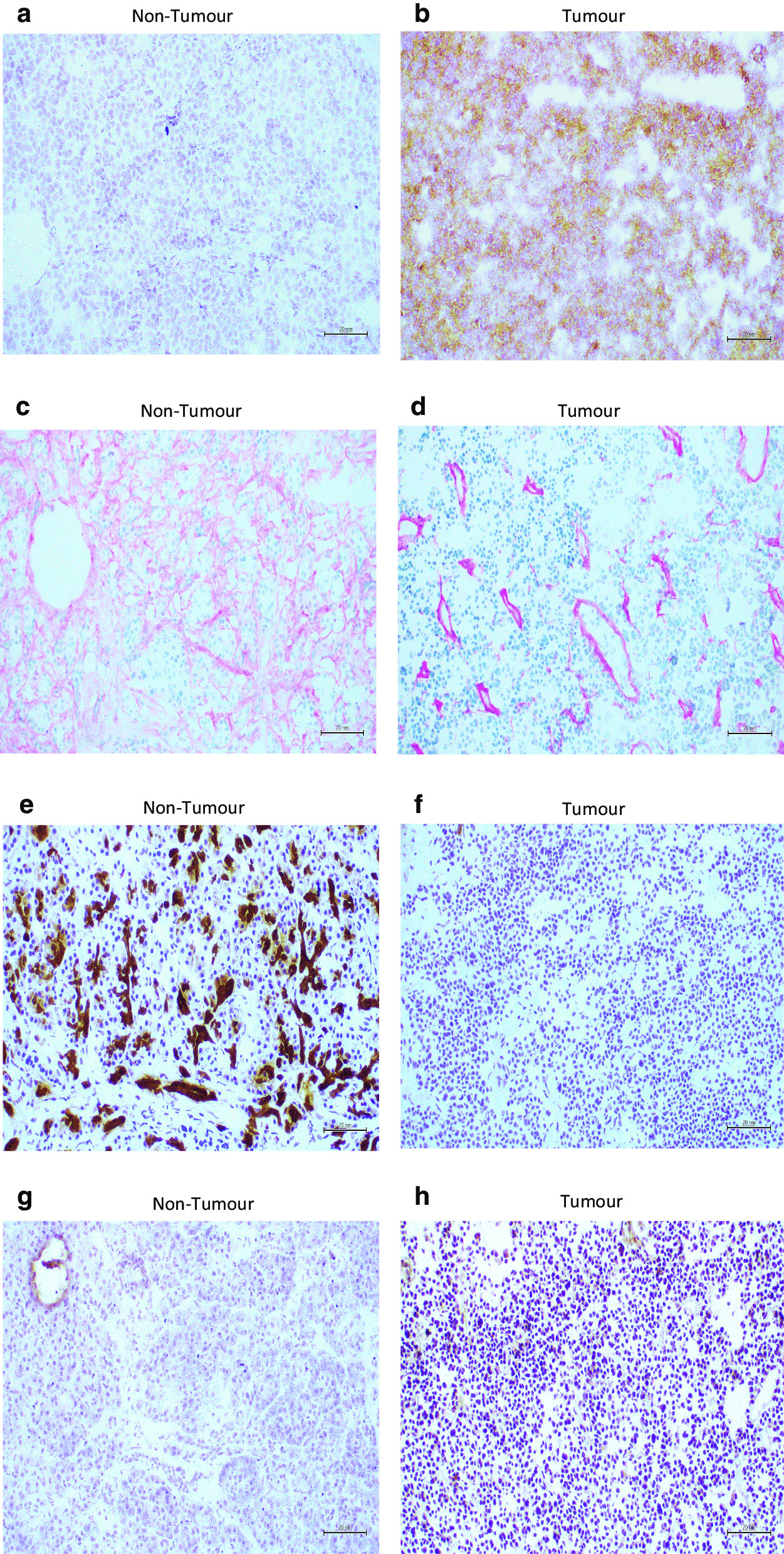
Immunohistochemistry images showing expression levels of β- catenin (**a**,**b**), Laminin (**c**,**d**), CK7 (**e**,**f**) and P53 (**g**,**h**) in pancreatic non-tumour and tumour tissue sections. (Scale bars = 20 µM)

The stem cell marker, CD24 showed no staining in non-tumour tissue and strong staining in all cells of the tumour tissue (Fig. 2e,f), while CD44 showed < 10% stained cells in non-tumour tissue and strong staining in most cells of the tumour tissue. The markers of angiogenesis CD31 (Fig. 2g,h) and CD45 were not detected in non-tumour tissue but showed focal staining associated with blood vessels. Cells in non-tumour tissue were non stained for the EMT markers vimentin and β-catenin (Fig. 3a,b) while MENA was found in a small proportion of cells in non-tumour tissue, and within the tumour tissue approximately 50% of cells were positive for vimentin and β-catenin, and had patchy staining for MENA.

Staining for the cell adhesion marker CD49 showed 10% positive cells in non-tumour tissue with less staining in tumour tissue. Laminin was strongly stained in the extracellular matrix in non-tumour tissue and in the tumour tissue the label was associated with blood vessels (Fig. 3c,d). ECAD staining was detected in < 25% of the cells in non-tumour tissue while the tumour tissue had 25–50% positive staining. PCAD was negative in both normal and tumour. EpCAM staining was found in 50% of cells in both non-tumour and tumour tissue.

No staining for the differentiation marker CDX2 was detected in either non-tumour or tumour tissue. PDX1 staining was detected in both non-tumour and tumour tissue. Strong staining for CD14 was detected in non-tumour tissue with less label in tumour tissue. CK6 stained approximately 40% of cells in non-tumour tissue and 20% of cells in tumour tissue. Both CK7 (Fig. 3e,f) and CK13 showed strong focal staining in non-tumour tissue with little or no staining in tumour tissue. CK20 staining was not detected in non-tumour tissue but over 50% of cells in tumour tissue were stained. Staining for the tumour suppressor P53 (Fig. 3g,h) was not detected in non-tumour tissue but was present in most cells within the tumour tissue.

### Comparison of immunohistochemistry and qPCR data

The differences in marker staining between tumour and non-tumour obtained from IHC were compared with mRNA marker expression levels from the qRT-PCR analysis of tumour and non-tumour tissue (Table 4). CgA, synaptophysin, NSE, CD56, CD45, vimentin, β -catenin, CD49, EpCAM, PDX1, CK6, CK7, CK13, CK20, P53 were consistent in both mRNA and protein levels between tumour and non-tumour tissue. Other markers including Ki67, PCNA, DAXX, CD24, CD44, CD31, MENA, Laminin, ECAD, PCAD and CDX2 and CK14 showed different trends in expression between tumour and non-tumour with qRT-PCR compared to IHC. CDX2 and PCAD showed decreased mRNA levels in tumour relative to non-tumour and an absence of these proteins in normal and tumour tissue.

**Table 4:** Comparison of marker expression levels in non-tumour and tumour tissue for IHC and mRNA

Markers whose PCR expression levels between non-tumour and tumour correlated with IHC	Markers whose PCR expression between non-tumour and tumour did not correlate with IHC
CgA	Ki67
Synaptophysin	PCNA
NSE	DAXX
CD56	CD24
CD45	CD44
Vimentin	CD31
β-catenin	MENA
CD49	Laminin
EpCAM	ECAD
PDX1	PCAD
CK6	CDX2
CK7	CD14
CK13	
CK20	
P53	

mRNA levels of Ki67, CD24, CD44, CD31, MENA and ECAD were reduced in tumour tissue relative to normal tissue, whereas the protein expression was increased in the tumour compared to normal tissue. mRNA levels of PCNA, DAXX, laminin and CD14 in tumour tissue were equal to the levels seen in the non-tumour tissue whereas protein expression was increased for PCNA and decreased for laminin and CD14 in the tumour tissue relative to the non-tumour tissue.

## Discussion

Limitations in the current tumour classification and grading for NEN subtypes have highlighted the lack of predictive and prognostic markers [16]. The routinely used immunochemical markers CgA, synaptophysin and CD56 confirm a neuroendocrine diagnosis and the proliferation marker Ki67 is used a key indicator for the grading of NENs [12]. These markers have some limitations including their lack of correlation to clinical outcomes and their presence in non-tumour tissue. The routinely used markers represent only a few of the molecules that characterise the tumour phenotype. Thus, additional markers are required to more comprehensively define NEN subtypes.

In this study we used quantitative real-time reverse transcription PCR (qRT-PCR) to measure the mRNA levels of twenty-seven cancer markers associated with proliferation, metabolic activity, invasive potential and metastasis, in tissue from a patient with a G1 pancreatic NET. Comparison of mRNA marker levels from tumour tissue with adjacent non-tumour tissue from the same patient provided novel insights into the changes associated with acquisition of the cancer phenotype. The inclusion of matched controls enabled a comparative analysis, in comparison to many previous studies reporting tumour data only, which has limited value by itself when the status of the non-tumour tissue is unknown. Of the twenty-seven markers, CgA had a transcript abundance that was 80 times higher in tumour relative to non-tumour. CD56 and p53 were 6.5-fold more abundant in tumour relative to non-tumour tissue and β -catenin, PDX1 and CK20 were 2–3 times as abundant in tumour than non-tumour tissue. The profile of the tumour tissue indicated an overall reduction in mRNA levels of the analysed markers relative to non-tumour tissue. Ki67, PCAD and CK7 expression was reduced up to tenfold in the tumour relative to non-tumour with 2—ninefold reductions in CD24, CD44, CD31, CD49 MENA, ECAD, EPCAM, CDX2, CK6 and CK13. This suggests an overall loss of activity of these genes in the tumour. The most distinctive features of the tumour tissue were a gain in CgA, CD56, p53, PDX1, CK20 and a loss of Ki67, PCAD and CK7.

The observed increases in mRNA transcripts of the mesenchymal markers vimentin and β -catenin, decreased transcripts of the cell adhesion markers E-cadherin and P-cadherin and loss of differentiation markers CDX2, CK6, CK7 and CK13 are hallmarks of transition to a mesenchymal phenotype tumour cells. The epithelial to mesenchymal transition is a process by which normal epithelial cells lose their cell:cell and cell:matirix adhesion and acquire the migratory and invasive characteristics of a cancer cell [18].

This study indicates that qRT-PCR can distinguish phenotypic differences between tumour and non-tumour tissue. Advantages of qRT-PCR are that it is a semi-quantitative tool that generates numerical values for marker expression so comparisons can be made between different patients, as each marker is measured relative to a housekeeping gene. Other benefits are that it is quick and multiple markers can be tested in a single run. qRT-PCR is cheap, requiring only a few enzymes, buffers and primers. The same set of identical primers can be used in multiple labs thereby reducing variabilities between labs. A further advantage of qRT-PCR is that the RNA is extracted from the whole tissue and same extract used for all marker analyses. The extract is representative of the whole tissue rather than a portion of the tissue. PCR avoids the use of an antibody and the variability associated with antibodies to the same protein but from different sources.

qRT-PCR measures mRNA levels markers relative to a housekeeping gene or control gene. This is selected to be stably expressed under different experimental conditions and for its expression to remain constant in different tissues and not influenced by the presence of disease so the expression of other genes can be measured relative to it. β-actin and GAPDH are commonly used housekeeping genes as their expression has been found to remain constant under different conditions [19, 20]. However, studies indicate that some housekeeping genes could be better than the others for a particular sample, as the expression of these and various other housekeeping genes can be different in different sample cohort. Hence, we tested the β-actin and GAPDH housekeeping genes.

and selected the one with least variation. We found β-actin to have less variation between tumour and non-tumour, compared to GAPDH for the pancreatic NET sample. Therefore, β-actin was selected as the housekeeping gene for analysis and future experiments.

To provide a profile of the different markers expressed in tumour and non-tumour tissue, we measured mRNA levels of markers in each of these tissues relative to each other using DAXX as a reference as this gene was expressed in similar levels in tumour and non-tumour. This data showing the pattern of expression of different markers in tumour and non-tumour tissue provides a profile that may have application in defining individual patients. CgA for example is seen to be significantly low in the normal tissue profile while it has a massive increase in the tumour tissue. Future studies are required to validate these data using a larger number of patients and determine whether such profiles have potential for stratification of NEN tumours. A larger cohort may establish the possibility to Identify markers that are evidently different between normal and tumour to draw out a reference profile for different grades of NETs.

We compared qRT-PCR results with IHC. Fifteen of the twenty-seven markers showed the same expression trends between tumour and non-tumour tissue and twelve showed differences in trends between tumour and non-tumour tissue. The most notable differences between the qRT-PCR and the IHC results were Ki67, MENA, ECAD and the cluster of differentiation markers CD24, CD44 and CD31. Our data showed Ki67 was higher in tumour relative to non-tumour using IHC but qRT-PCR analysis indicated Ki67 mRNA was reduced in tumour tissue relative to the non-tumour. Previous studies comparing Ki67 protein with Ki67 mRNA levels indicated variable correlations amongst different tumour samples [21]. Variability in Ki67 expression has been attributed to changes in Ki67 levels with different stages of the cell cycle [22]. It is possible that the low levels of Ki67 found in our study may correspond with regions of the tumour containing non-cycling cells, given that IHC only utilises a small proportion of the total tumour mass. qRT-PCR for Ki67 was found to be more accurate than IHC in a breast cancer study in predicting patient response [23]. ECAD expression associated with breast cancer metastasis is understood to be regulated by cell surface integrin α3β1. α3β1 inversely affects the mRNA expression level of ECAD and this is counter balanced by the protein expression [24].

Our study showed that for four of the seven CD markers (CD24, CD44, CD31, CD14), the mRNA and protein data did not correlate. This is consistent with a previous study where concordance between gene and protein expression for a range of CD antigens in normal and prostrate tumour samples was poor to moderate (Pearson correlations ranged from 0 to 0.63), attributed to low levels of protein expression, sample preparation as well as the real biological differences between protein and mRNA expression [25]. CD24 functions in cell adhesion and signaling, where high expression is associated with increased proliferation and invasion in pancreatic, colorectal and lung cancer but decreased proliferation and invasion in breast cancer cells [26]. The lack of direct correlation between mRNA and protein levels may be related to the complex regulatory pathways between CD24 transcription and translation. CD31 is an endothelial marker used as an indicator of blood vessels. Its expression may relate to the amount of vascular tissue which can vary considerably depending on the microscopic fields selected for IHC.

Synaptophysin was not detected by IHC and was only barely detectable by qRT-PCR, showing a slight increase in tumour relative to non-tumour. The levels of this transcript may not have been sufficient to produce detectable levels of protein. IHC staining for synaptophysin varies between different NENs and was reported as positive for approximately 60% of gastrointestinal neuroendocrine carcinomas [27]. EPCAM mRNA levels were lower in the tumour tissue than non-tumour and the IHC label was also lower in tumour relative to non-tumour however both tumour and non-tumour were categorised as 1–25% stained and not sufficiently different to be distinguishable. CD49 showed less mRNA in tumour than non-tumour. This pattern was also observed in IHC where staining was slightly less in tumour than non-tumour but both tumour and non-tumour showed < 25% positive staining.

IHC is the traditional method for tumour diagnosis, essential for establishing tissue morphology and providing information for grading of tumour biopsies. IHC provides information about morphology of tissue that cannot be obtained with PCR. IHC is a qualitative and relatively time-consuming procedure in which the scoring technique may dependent on the scorer. Small changes of a few percentage in the Ki67 index can affect the grading of the tumour which itself may have an influence on the type of treatment regime chosen. While the new WHO NEN guidelines have improved the stringency of IHC methods [12], the heterogeneity of tissue, differences in preparation of tissue, differences in counting approaches (automated versus manual) as well as variations in the sensitivity and specificity of antibodies adversely affects the prognostic potential of IHC [28]. IHC requires further development to become an immunoassay, not simply a stain [29]. Computed tomography however, has successfully been used to characterise intraductal calculi and pancreatic calcifications in chronic pancreatitis [30]. This technology may have potential for application in cancers such as NENs.

## Conclusion

Our study provides a unique analysis for NEN tissue relative to other reported studies, through utilization of the technique of PCR and inclusion of control tissue for comparison to the tumour tissue. The panel of markers screened includes a wide range that encompasses many tumour characteristics. PCR analysis of a G1 pancreatic sample revealed differential expression of 20 markers between tumour and matched normal tissue, out of a total of 27 markers tested. That many markers were found to be differentially expressed between tumour and non-tumour indicates marker profiling as a potential, additional diagnostic tool for better defining and stratification of tumours. As this was a pilot study based on a single NET case, the utility of the approach to measure multiple markers requires validation in a cohort study of different NEN grades and correlation with clinical outcomes. A better understanding of tumour cell phenotypes and their cell biology will contribute to improved clinical outcomes for patients.

## Supplementary Information

**Additional file 1:** Detailed materials and methods.

## Data Availability

All qRT-PCR data and immunohistochemistry images are available from Prof M Leigh Ackland (leigha@deakin.edu.au).

## Abbreviations

NETs: Neuroendocrine tumours
NENs: Neuroendocrine neoplasm
NEC: Neuroendocrine carcinoma
IHC: Immunohistochemistry
qRT-PCR: Real-time quantitative PCR
CgA: Chromogranin A
NSE: Neuron Specific Enolase
CK: Cytokeratin
EMT: Epithelial to Mesenchymal Transition
