# Supplementary material for: mRNA profiling of a well-differentiated G1 pancreatic NET correlates with immunohistochemistry profile: a case report
Source: BMC Gastroenterol. 2021 Apr 27;21:194. doi: 10.1186/s12876-021-01705-9 (PMC8080317; doi:10.1186/s12876-021-01705-9)
Supplement: Supplementary file 1 — Additional file 1: Detailed materials and methods. [file 12876_2021_1705_MOESM1_ESM.docx]

**Methods**

*Sample Collection:*

The pancreatic tumour with its matched adjacent non-tumour tissue was obtained from the Victorian Cancer Biobank (VCB) and stored at -80^o^C until use.

*Experimental methods*

Quantitative Real-Time Reverse Transcription PCR (qRT-PCR)

RNA Extraction:

A 2mm X 2mm frozen tissue was cut and ground using mortar and pestle in the presence of liquid nitrogen and further homogenised by passing through 20 gauge needle and syringe multiple times. RNA was extracted from the tissue samples using the RNeasy mini kit (Qiagen) as per manufacturers instruction. RNA concentrations were measured using the Thermo Scientific NanoDrop 1000. Only RNA that passed the QC of 260/280 ratio of 2 was reverse transcribed into cDNA.

Reverse Transcription:

RNA was converted into cDNA using Applied Biosystems High-Capacity cDNA Reverse Transcription Kit as per manufacturer’s instruction. A working solution of 5ng/ul was prepared by diluting the stock using nuclease free water.

Real-Time PCR:

10ul of PowerUp SYBR Green Master Mix (Applied Biosystems), 5ng of cDNA template, 250nM of each primer was made up to 20ul in a PCR reaction/well using nuclease free water. Template (20ng) was used for markers which were expressed in very low levels. GAPDH and β-actin were used as the endogenous control. Every run also included negative controls of master mix without cDNA to rule out for contamination. The PCR cycle was 50 ^o^C- 2 min, 95^o^C- 10min and 45 cycles of 95 ^o^C- 15s, 60 ^o^C- 1min and a final extension for 95 ^o^C-15s. The plate was loaded on to Biorad CFX96 and the marker levels were analysed using relative quantification method (ΔΔCt). All markers were tested for 3 individual qRT-PCR runs and triplicate experiments were run for each sample. The fold change was calculated on excel and two tailed T test was used to determine the statistical significance.

Statistics:

T-test:

Two-tailed t-test with unequal variance was used to find the probability value (p-value) between each normal and tumour sample and its positive and negative standard deviation values. P-values less than 0.05 were concluded to be statistically significant.

One-way ANOVA:

For tissue specific marker profiling, expression levels of all markers in each of normal and tumour tissue was calculated using expression of DAXX as control. One-way ANOVA with post hoc tukey test was used to study the significance of the markers in each profile. Markers with a p value <0.05 in comparison to DAXX expression was identified to be significantly different in expression.

Immunohistochemistry (IHC):

Tissue Sectioning: A 3mm X 3mm tissue sample cut and a block prepared using OCT then frozen in liquid nitrogen. Sections of 5-8um thickness were sectioned and collected on a charged slide using a Thermo Scientific HM525NX cryostat. The slides were air dried and stored in -80^o^C until use.

Immunostaining:

Frozen sections were rinsed three times with 1XPBS for 10 minutes then fixed in 4% paraformaldehyde (PFA). Slides were washed three times with 1X PBS then incubated in 0.3% hydrogen peroxide for 10 min followed by 10 min incubation with 0.1% triton X with three 1X PBS washes between the two incubations. The sections were blocked in 2% BSA for 45 min then incubated with primary antibodies at 4^o^C overnight. The following day the slides were washed using 1X PBS and incubated with horse radish peroxidase or alkaline phosphatase enzyme tagged secondary antibody for 30 minutes. The corresponding substrate, ImmPACT DAB or alkaline phosphatase (Vector Laboratories) was then added, followed by counterstaining with methyl green or haematoxylin and mounting using DPX solution (Sigma Aldrich, Australia).

Imaging:

Images were taken at 40X, 100X and 400X magnifications using an Olympus BX43 light microscope fitted with a DP21 camera. Images were collected and analysed by comparing the intensity of label in the tumour sample in comparison to that seen in the matched normal tissue.
